# Supplementary figures and images for: Evaluation of parameters affecting performance and reliability of machine learning-based antibiotic susceptibility testing from whole genome sequencing data
Source: PLoS Comput Biol. 2019 Sep 3;15(9):e1007349. doi: 10.1371/journal.pcbi.1007349 (PMC6743791; doi:10.1371/journal.pcbi.1007349)

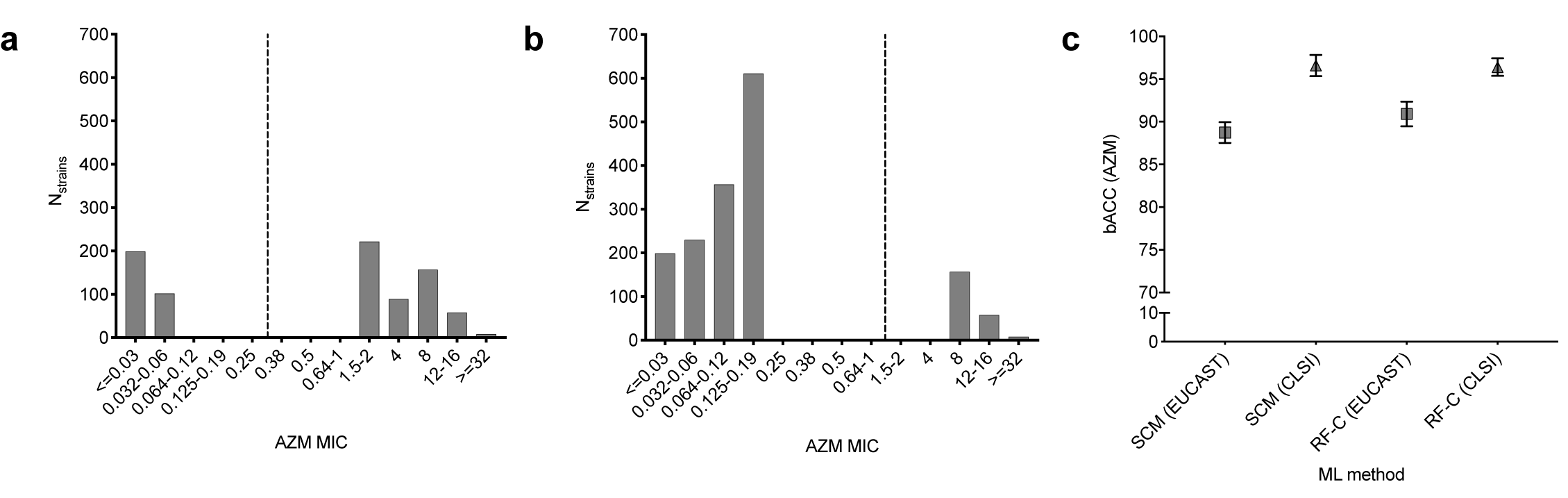

Supplement: S1 Fig — Histograms showing azithromycin (AZM) minimum inhibitory concentration (MIC) distributions for the aggregate gonococcal dataset after down-sampling to remove all strains with MICs ≤2 doubling dilutions of the (a) EUCAST or (b) CLSI breakpoint. (c) Mean balanced accuracy (bACC) with 95% confidence intervals of SCM RF-C predictive models trained and tested on down-sampled aggregate gonococcal datasets. (TIFF) [file pcbi.1007349.s008.tiff]

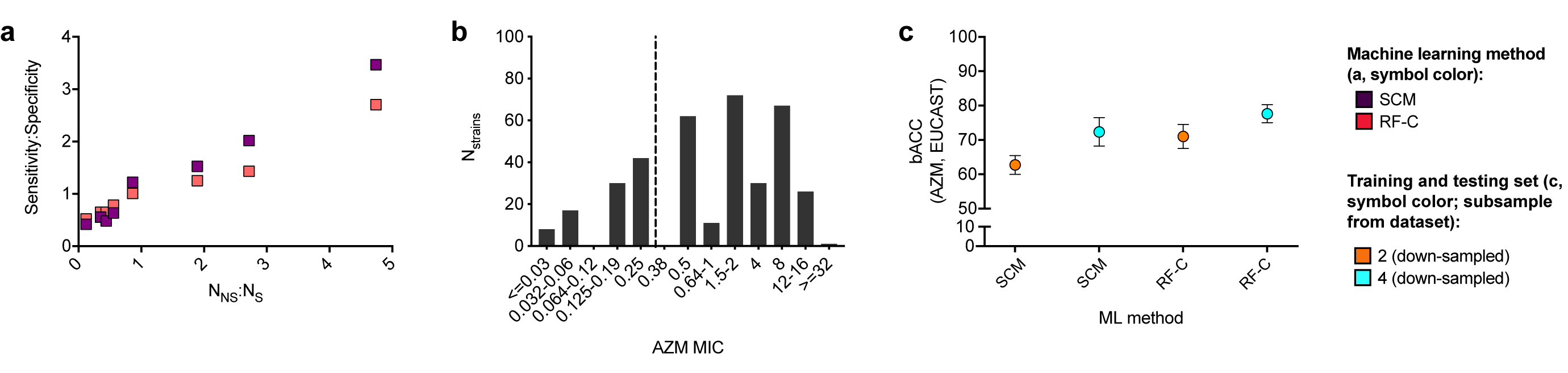

Supplement: S2 Fig — (a) Scatter plot showing the relationship between the ratio of azithromycin (AZM) non-susceptible (NS) strains to susceptible (S) strains (by the EUCAST breakpoint) in each dataset and the ratio of sensitivity to specificity achieved by set covering machine (SCM) and random forest binary classification (RF-C) methods. (b) Histogram showing the AZM minimum inhibitory concentration (MIC) distribution for both datasets 2 and 4 after down-sampling to equalize number of strains and MIC distributions between datasets. (c) Mean balanced accuracy (bACC) with 95% confidence intervals of RF-C predictive AZM NS models trained and tested on down-sampled datasets 2 and 4. Symbol colors in (a) indicated the machine learning (ML) method. Symbol colors (b) indicate the down-sampled dataset from which the training and testing sets were derived. (TIFF) [file pcbi.1007349.s009.tiff]

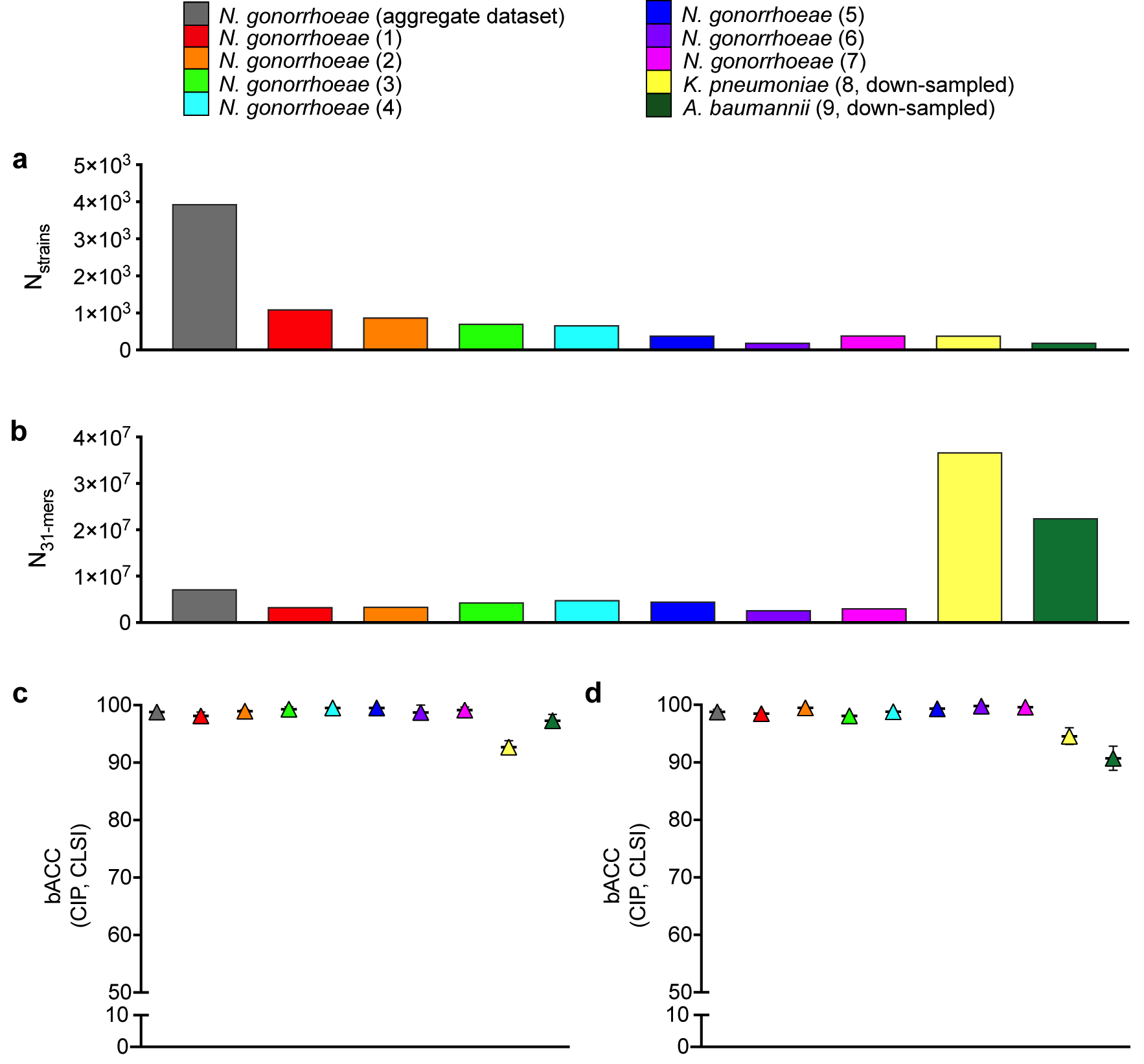

Supplement: S3 Fig — Number of (a) strains and (b) unique 31-mers present in the genomes of at least two strains in each dataset, after down-sampling the K. pneumoniae and A. baumannii datasets to equalize the number of S and NS strains within each dataset. Mean balanced accuracy (bACC) with 95% confidence intervals achieved by (c) set covering machine and (d) random forest classification models for ciprofloxacin (CIP) NS by the CLSI breakpoints across gonococcal, down-sampled K. pneumoniae, and down-sampled A. baumannii datasets. (TIFF) [file pcbi.1007349.s010.tiff]
